# Rare phylotypes in stone, stool, and urine microbiomes are associated with urinary stone disease

**DOI:** 10.3389/fmolb.2023.1210225

**Published:** 2023-08-04

**Authors:** Mangesh Suryavanshi, Jose Agudelo, Aaron Miller

**Affiliations:** ^1^ Department of Cardiovascular and Metabolic Sciences, Cleveland Clinic, Cleveland, OH, United States; ^2^ Department of Urology, Glickman Urological and Kidney Institute, Cleveland Clinic, Cleveland, OH, United States

**Keywords:** rare phylotype, urinary stone disease, human microbiome, urology, urobiome, reference database, kidney stones

## Abstract

**Introduction:** In complex microbial communities, the importance of microbial species at very low abundance levels and their prevalence for overall community structure and function is increasingly being recognized. Clinical microbiome studies on urinary stone disease (USD) have indicated that both the gut and urinary tract microbiota are associated with the onset of the disease and that kidney stones them-selves harbor a complex, yet consistent and viable, microbiome. However, how rare phylotypes contribute to this association remains unclear. Delineating the contribution of rare and common phylotypes to urinary stone disease is important for the development of bacteriotherapies to promote urologic health.

**Methods:** The objectives of the current report were to conduct a metaanalysis of 16S rRNA datasets derived from the kidney stone, stool, and urine samples of participants with or without urinary stone disease. To delineate the impact of rare and common phylotypes, metaanalyses were conducted by first separating rare and common taxa determined by both the frequency and abundance of amplicon sequence variants.

**Results:** Consistent with previous analyses, we found that gut, upper urinary, and lower urinary tract microbiomes were all unique. Rare phylotypes comprised the majority of species observed in all sample types, with kidney stones exhibiting the greatest bias toward rarity, followed by urine and stool. Both rare and common fractions contributed significantly to the differences observed between sample types and health disparity. Furthermore, the rare and common fractions were taxonomically unique across all sample types. A total of 222 and 320 unique rare phylotypes from urine and stool samples were found to be significantly associated with USD. A co-occurrence correlation analysis revealed that rare phylotypes are most important for microbiome structure in stones, followed by urine and stool.

**Discussion:** Collectively, the results indicate that rare phylotypes may be important for the pathophysiology of USD, particularly in the kidney stone matrix, which is inherently a very low microbial biomass niche that can have implications for the diagnosis and treatment of kidney stones. Further studies are needed to investigate the functional significance of rare phylotypes in kidney stone pathogenesis.

## Introduction

Urinary stone disease affects millions of people worldwide, and recent studies have highlighted the potential role of the microbiome in the pathogenesis of this condition (Kachroo et al., 2021; Miller et al., 2022). Ecological rarity, characterized by low abundance or limited distribution, is common among most species, yet our understanding of the factors that contribute to the persistence of rare species and how they contribute to the microbial community structure and function remains limited (Magurran and Henderson, 2003; Violle et al., 2017). With the advent of next-generation sequencing technologies, rare microbial taxa and the interactions between rare taxa are more easily studied; a broader microbial community in which they inhabit and their environment can also be revealed. Here, we propose that rare taxa play a critical role in maintaining the ecological balance in the human microbiome and provide an outstanding contribution to the structure of very low microbial biomass communities, such as those found in kidney stones.

Metagenomic sequencing of stool, urine, and stone samples can be used to analyze gut-, urinary-, and stone-associated microbiomes, respectively. Rare phylotypes have been identified as potentially important modifiers of disease risk, but methods for their segregation and statistical analysis are still in their infancy (Price et al., 2010; Zhang et al., 2010). Therefore, a standardized method for analyzing rare phylotypes is needed for the better understanding of their potential implications for disease diagnosis and treatment.

In this study, we conducted a meta-analysis of 16S rRNA amplicon sequence data from six different studies (SRP140641, SRP140933, SRP066940, SRP103884, SRP125171, and SRP125191) to identify rare phylotypes that are consistently associated with urinary stone disease. Our goal was to delineate the contributions of rare and common phylotypes to urinary stone disease and to explore the potential diagnostic and therapeutic implications of these findings. By pooling data from multiple studies and analyzing them using a common methodology, we aim to provide a more comprehensive and accurate assessment of the microbiome associated with urinary stone disease. Moreover, through the establishment of selection criteria for rare taxa from quality-controlled sequencing data, future research studies can expand upon the impact of rare taxa in the microbiome.

## Materials and methods

Raw data for the meta-analysis were downloaded from the respective Sequence Read Archive (SRA) accession numbers SRP140641, SRP140933, SRP066940, SRP103884, SRP125171, and SRP125191 for independent data analysis that builds on previous meta-analyses (Kachroo et al., 2021). Quality control and taxonomic assignment of sequencing data retrieved from the SILVA 138 SSURef and NCBI databases were performed using the DADA2 pipeline (R statistical package) (Callahan et al., 2016) to assign taxonomy to the amplicon sequence variants (ASVs), as previously described (Kachroo et al., 2021). Briefly, to eliminate sequencing artifacts and errors, quality filtering, trimming, and bimera removal were performed in DADA2 using default parameters. Unclassified sequences and those classified as eukaryotes, mitochondria, or chloroplasts were removed from further analysis using the phyloseq (McMurdie and Holmes, 2013) package in R (R-Project, 2023). Data were processed using commonly used pipelines (Kachroo et al., 2021). However, to delineate the contributions of rare vs*.* common phylotypes to US, data were then separated into rare and common phylotypes as follows: rare phylotypes were defined as ASVs with a prevalence and total sequence count below the average value for all ASVs in that sample type, whereas common phylotypes were ASVs with a prevalence and sequence count above the average value for both metrics.

Alpha and beta diversity analyses, ASV distribution, taxonomic profiling, and differential abundance analysis were performed using phyloseq and vegan packages (Oksanen et al., 2010), along with base R. Alpha diversity was calculated as phylogenetic diversity, while beta diversity was calculated as a weighted UniFrac dissimilarity index (Lozupone et al., 2006). Differential abundance analysis was performed using the DESeq2 package in R (Love et al., 2014), which models count data from sequencing experiments and tests for differential abundance using a negative binomial distribution. The results of the differential abundance analysis were visualized using ggplot2, and ternary plots were generated using the Ternary package (Smith, 2017). The metadata used for the analyses are provided in Supplementary Material S1.

Co-occurrence networks were calculated through all pairwise correlations of ASVs within the stool, urine, and kidney stone datasets in base R. Correlations with a false discovery-corrected *p*-value <0.05 and r >0.4 were considered significant.

## Results

**FIGURE 1:**
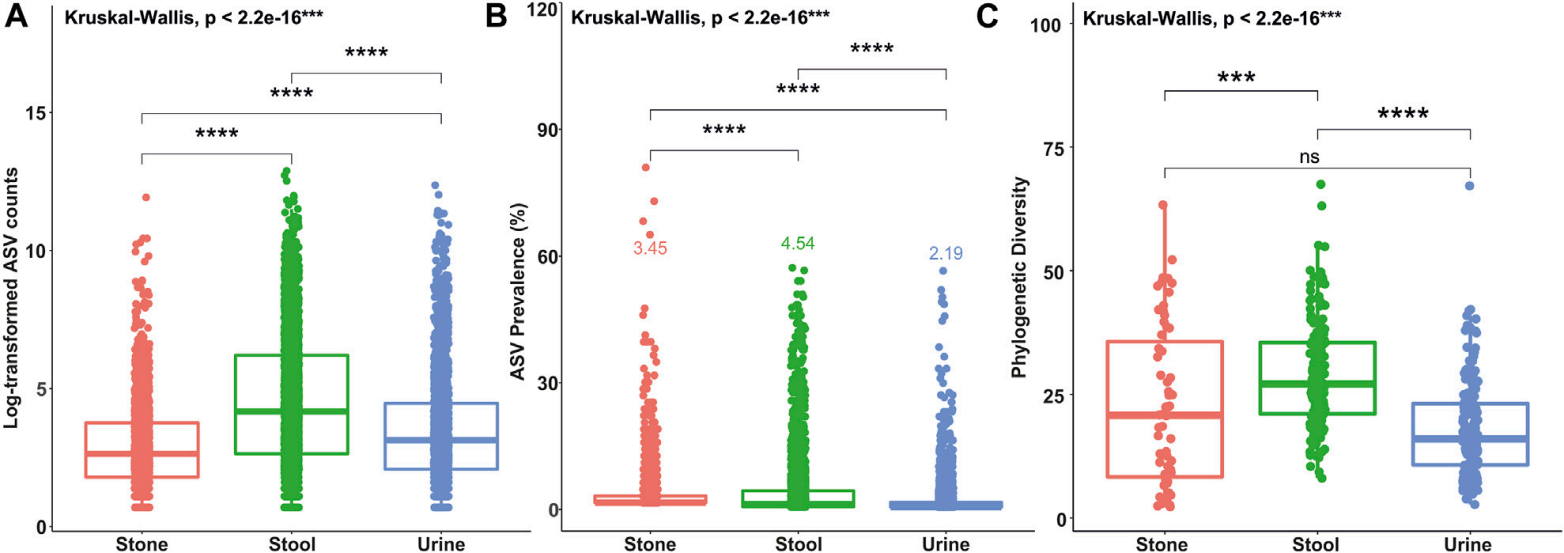
Box plots showing differences in ASV counts **(A)**, ASV prevalence **(B)**, and phylogenetic diversity indices values **(C)** between the stone, stool, and urine samples. Statistical differences were calculated based on the Kruskal–Wallis test between the groups and Holm’s corrected, paired *t*-tests. ****p <0.0001; ***p <0.001; **p <0.01; *p <0.05; ns, not significant.

A total of 201 samples from USD patients and 136 samples from healthy controls were analyzed for this study with no significant heterogeneity in alpha and beta diversity results between the studies (Kachroo et al., 2021). There were 39,295±1,222 sequences per sample. The average ASV prevalence was 2.19%, 3.45%, and 4.54% in the urine, stone, and stool samples, respectively. The average number of sequence counts per ASV was 927, 253, and 2,201 in the urine, stone, and stool samples, respectively. Defining rare and common ASVs as being below or above both prevalence and abundance thresholds covered 99.6%, 99.3%, and 98.4% of all ASVs in urine, stone, and stool samples, respectively. Of those, 7.5%, 5.1%, and 10.9% of ASVs were common in urine, stone, and stool samples, while the remaining were identified as rare. The distribution of ASVs by the sequence count and prevalence are presented in Supplementary Figures S1, S2. A Kruskal–Wallis test revealed that the average ASV prevalence and sequence count in each sample type was significantly different from each other (Figures 1A, B). The taxonomic breakdown at the phylum and genus levels for common and rare taxa in stone, stool, and urine samples is presented in Supplementary Figures S3–S5.

### Microbiome diversity in stone, stool, and urine samples

**FIGURE 2:**
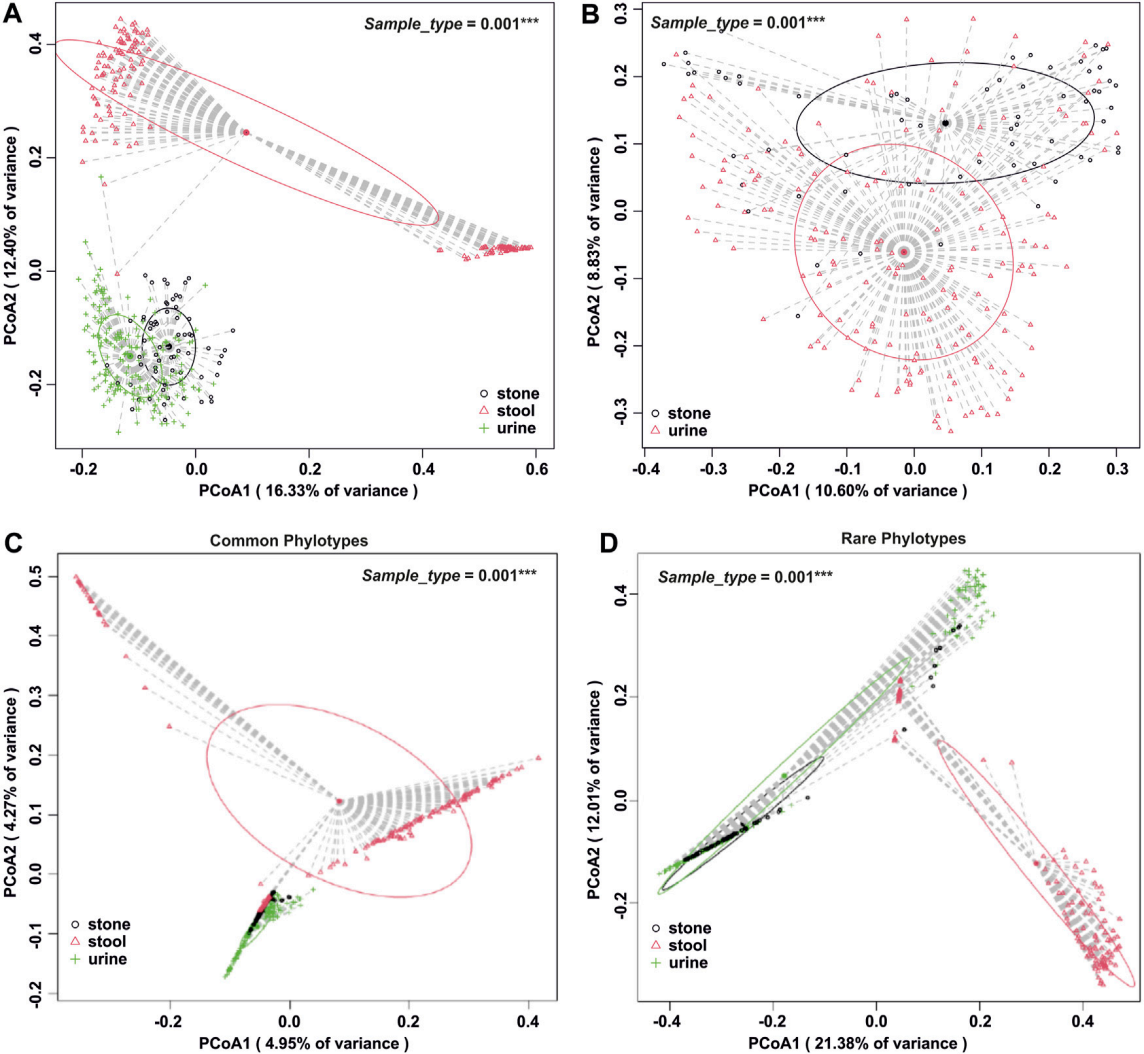
Beta diversity analysis by the sample type. **(A)** Whole-microbiome analyses for all sample types. Global *p* = 0.001 and all paired *p* = 0.002 after Holm’s correction. **(B)** Isolation of beta diversity analysis for urinary tract samples, *p* = 0.001. **(C)** Beta diversity analysis of all sample types, isolated to the common fraction of the microbiome. Global *p* = 0.001 and all paired *p* = 0.002 after Holm’s correction. **(D)** Beta diversity analysis of all sample types, isolated to the rare fraction of the microbiome. Global *p* = 0.001 and all paired *p* ≤0.003 after Holm’s correction. Beta diversity analysis was carried out using an unweighted UniFrac dissimilarity matrix, and statistical analysis was conducted as a one-way global or paired PERMANOVA with 999 permutations.

The number of phylogenetic clusters was overall significantly higher in stool samples compared to urine and stone samples (Figure 1C). An unweighted UniFrac analysis, which considers the presence/absence of phylogenetic clusters, revealed that all sample types were unique from each other (Figures 2A, B; *p*-value = 0.002 for all pairwise comparisons). To determine whether rare or common phylotypes drive the differences between sample types, we conducted an unweighted UniFrac comparison of common or rare phylotypes between sample types and found that both common and rare taxa contribute to the differences observed in the microbiome composition between all sample types. Although the rare phylotypes led to an overall greater separation of communities, based on the percent variance separating the groups in the PCoA (*p*-values: urine vs*.* stone = 0.003; urine vs*.* stool = 0.002; stone vs. stool = 0.002), common phylotypes also led to a significant separation between sample types (*p*-value = 0.002 for all pairwise comparisons). Beta diversity results were similar to those of weighted UniFrac analyses, which show that the differences observed are driven by the presence/absence of taxa and not just relative abundance. These data indicate that both common and rare taxa contribute to the differences observed in the microbiome composition between sample types (Figures 2C, D).

All sample types exhibited greater taxonomic diversity in the rare phylotypes compared to common phylotypes (Supplementary Figures S3–S8). However, while the rare phylotypes in the stone and urine samples were greatly depleted in Firmicutes compared to those in the common fraction (Supplementary Figures S3, S7), the opposite was true for stool samples (Supplementary Figure S5). Importantly, the common fraction in the stone microbiome was dominated by *Escherichia coli* (Supplementary Figure S4A), which is a known uropathogen that has previously been reported as one of the most common and abundant pathogens found in the stone microbiome (Dornbier et al., 2019; Zampini et al., 2019; Kachroo et al., 2021).

**FIGURE 3:**
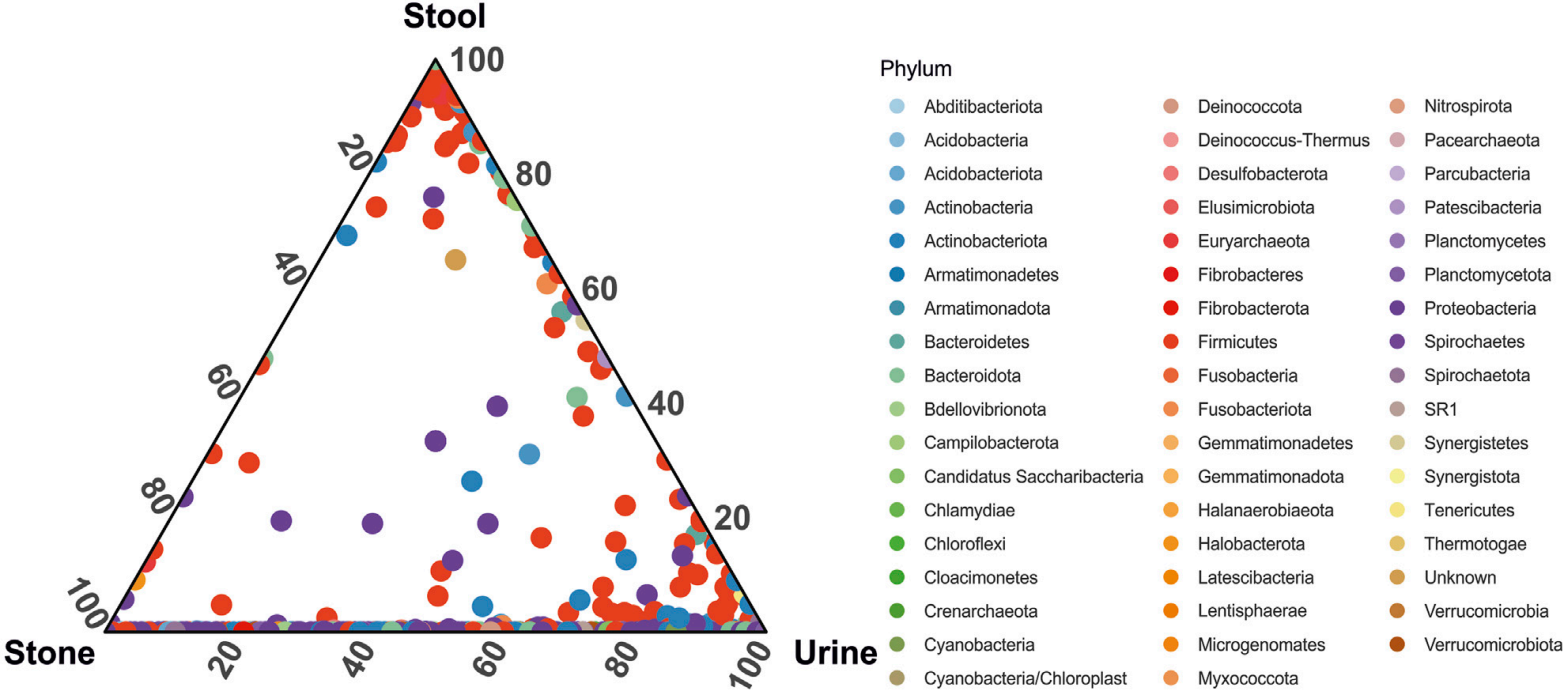
Ternary plot representing the relative abundance of individual ASVs (circles) in the stone, stool, and urine samples. Taxa are colored by phylum-level taxonomy.

When looking at the relative abundance of ASVs common to each sample type, we see that although the overall microbiome composition is significantly different, urine and stone microbiomes exclusively share a considerable number of ASVs overall (Figure 3). There are far fewer shared ASVs between stool and urine samples and almost no shared ASVs between stone and stool samples (Figure 3). Approximately 25 ASVs, primarily from the Proteobacteria and Firmicutes phyla, were common in all sample types.

### Rare phylotypes provide the overall microbiome structure

**FIGURE 4:**
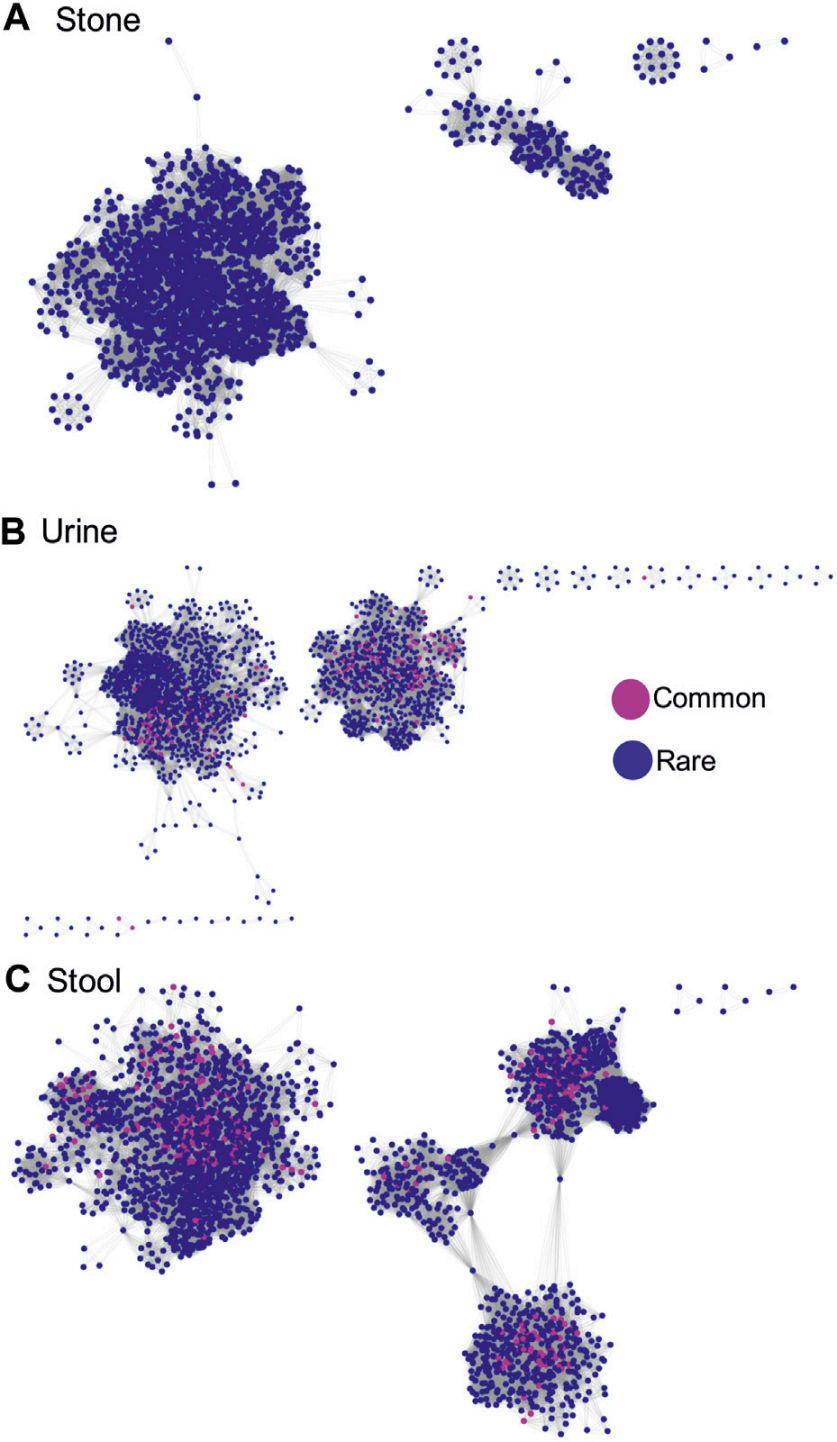
Correlation networks showing inter-phylotype (based on ASVs) positive Pearson’s correlations within sample types. Correlations are based on the false discovery rate-corrected *p*-values <0.05 and correlation coefficients >0.4 from all pairwise comparisons of ASVs. ASVs were labeled as either common or rare. A positive correlation occurs when the abundance of one ASV increases and decreases with another ASV across all the samples analyzed. **(A)** Network showing positive associations between common and rare phylotypes in stone samples. **(B)** Network showing positive associations between common and rare phylotypes in stool samples. **(C)** Network showing positive associations between common and rare phylotypes in urine samples.

To determine the importance of rare vs*.* common phylotypes for the overall community structure, we conducted a Pearson’s correlation analysis based on the false discovery rate-corrected *p*-values <0.05 and correlation coefficients of >0.4 from all pairwise comparisons of ASVs within each sample type. Positive correlations, shown here, occur when the abundance of one ASV increases and decreases with another ASV across all analyzed samples. The assumption is that since the populations increase and decrease together, the paired ASVs are somehow dependent on each other. When we visualize the resulting correlation networks, we see that, in stone samples, one major and a few minor microbial networks are formed, which are completely dominated by rare phylotypes (Figure 4A). In contrast, both urine and stool samples had two major networks, both of which had common phylotypes central to the clusters that were formed (Figures 4B, C). These data corroborate all of the previous data, which again suggests that rare phylotypes are most important for stone samples, followed by urine and stool samples.

### Microbiome diversity through health disparity

**FIGURE 5:**
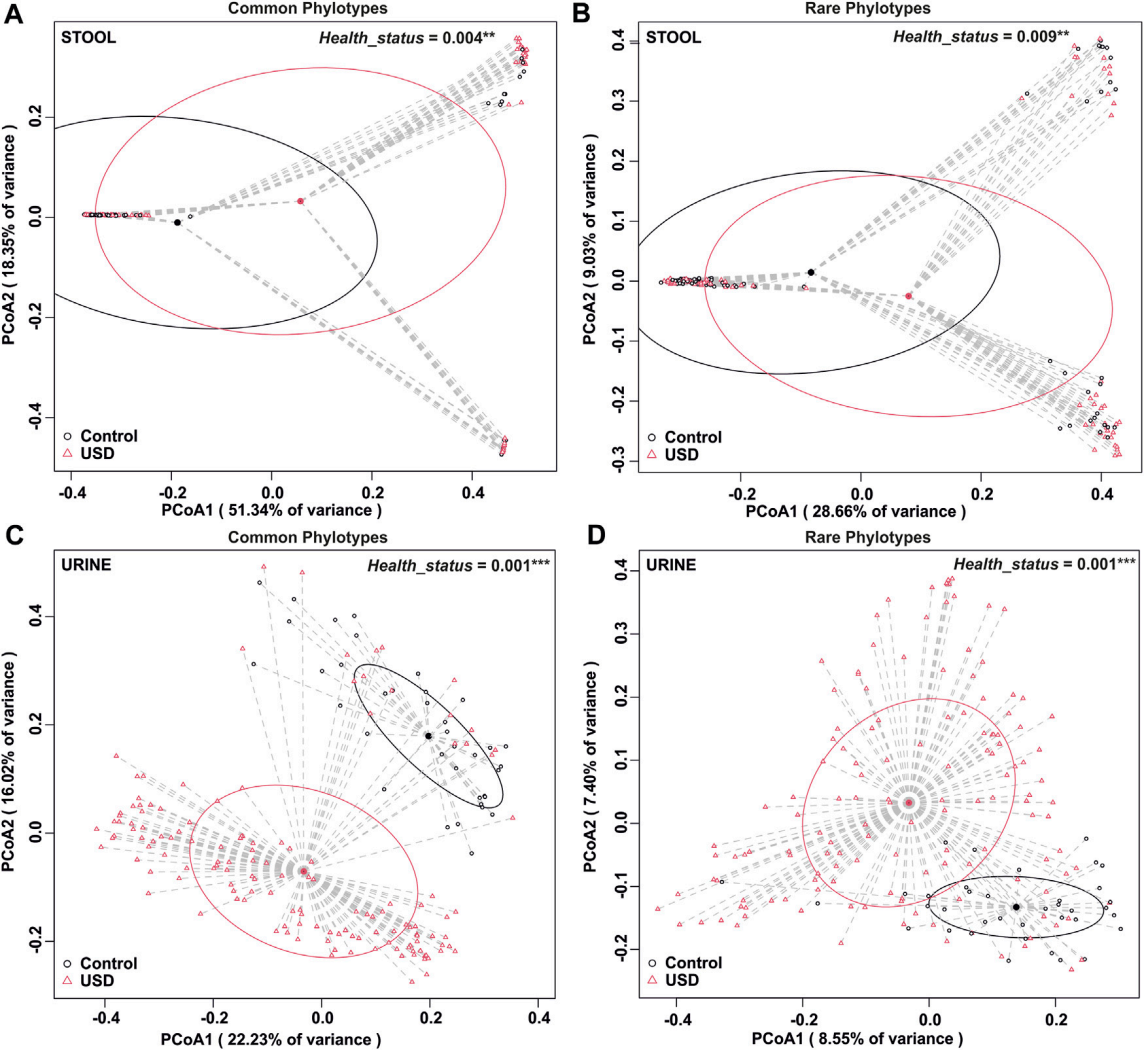
Beta diversity analysis by health disparity in urine and stool samples. **(A)** Analysis of the common fraction in stool samples, *p* = 0.004. **(B)** Analysis of the rare fraction in stool samples, *p* = 0.009. **(C)** Analysis of the common fraction in urine samples, *p* = 0.001. **(D)** Analysis of the rare fraction in urine samples, *p* = 0.001. Beta diversity analysis was carried out using an unweighted UniFrac dissimilarity matrix, and statistical analysis was conducted as a one-way global or paired PERMANOVA with 999 permutations.

To determine whether rare or common phylotypes differentiate gut and/or urine microbiota through health disparity (healthy or USD), we conducted an unweighted UniFrac comparison of common or rare phylotypes of stool and urine samples between healthy controls and USD patients (Figure 5). Here, while the common phylotypes lead to a greater separation of microbiome compositions, as determined by the percent variance separating the groups in PCoA, all comparisons were significantly different (*p* <0.01), which indicates that both rare and common phylotypes in both gut microbiota and urinary tract microbiota are associated with kidney stone disease.

**FIGURE 6:**
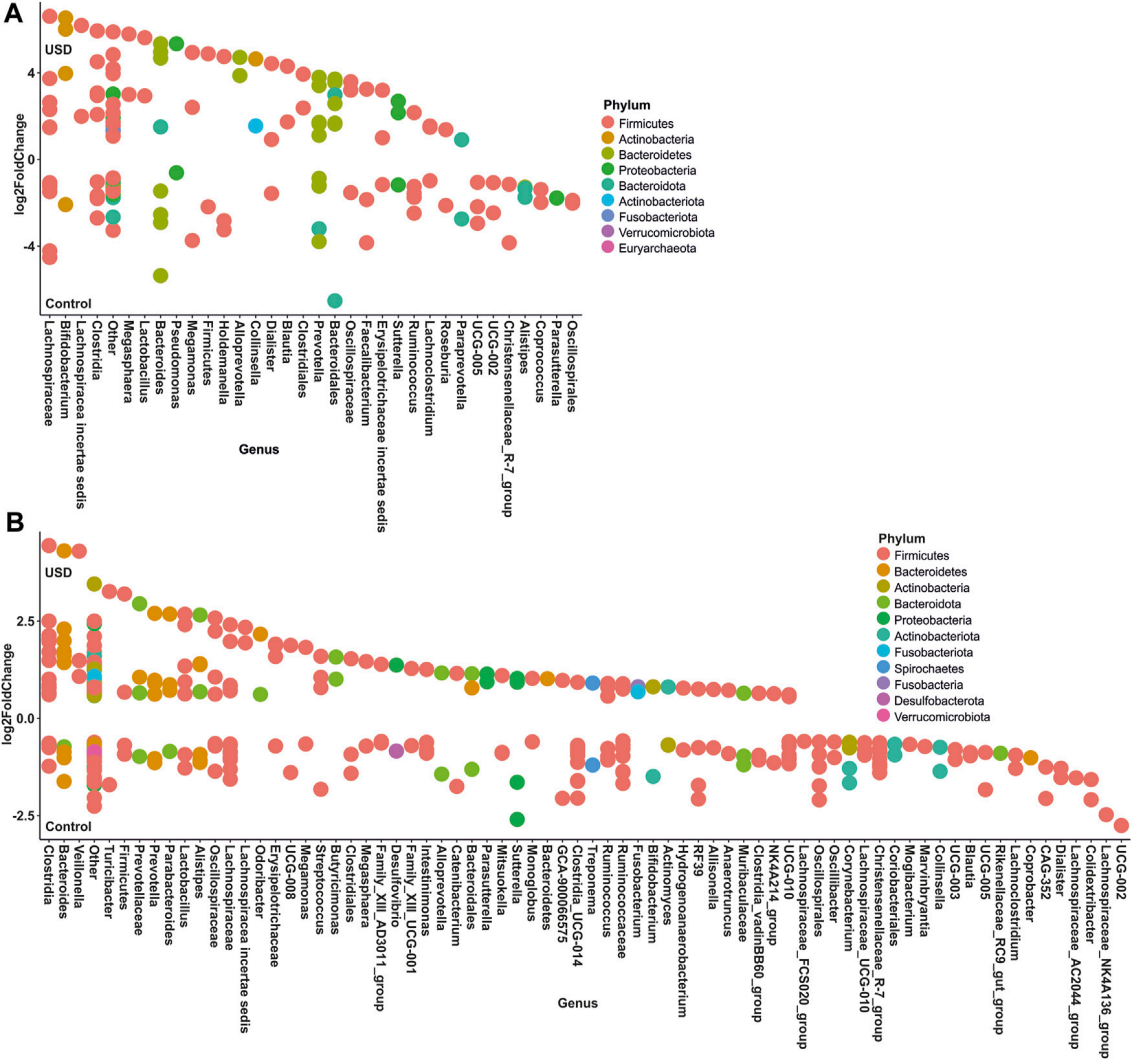
Bubble plot showing the fold change of differentially abundant common **(A)** and rare **(B)** taxa associated with health disparities (control and USD phenotypes) in stool samples. Selection of taxa is defined using DESeq2 differential abundance analysis with a false discovery rate-corrected *p*-value <0.05. Taxa are listed as the number of ASVs within the lowest assigned taxonomy as a means to elucidate the most important taxa associated with USD.

**FIGURE 7:**
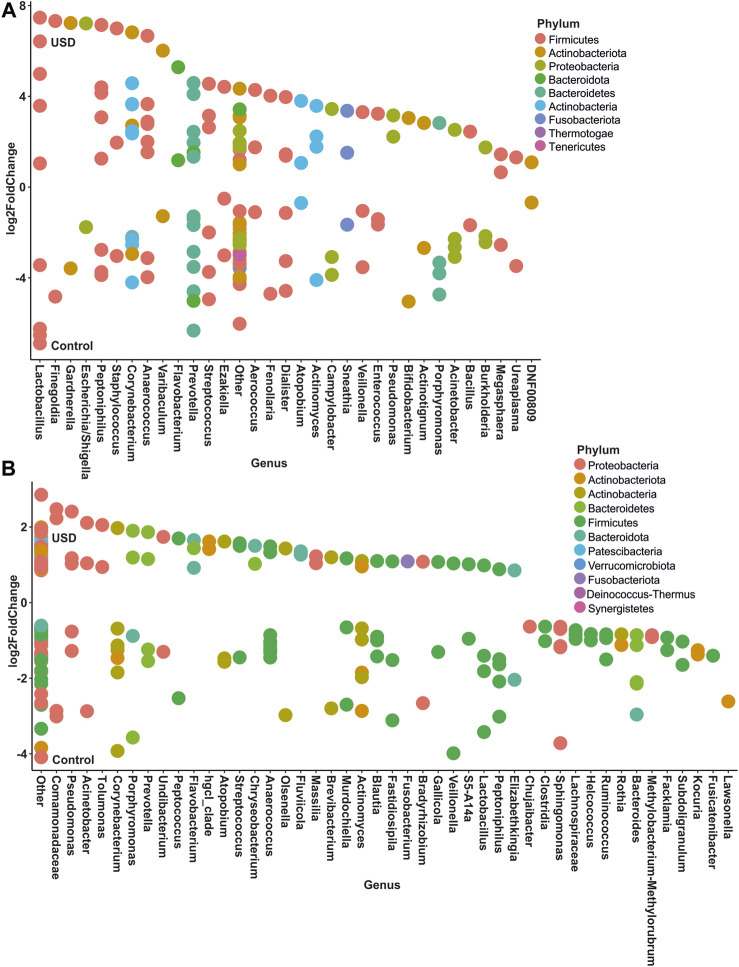
Bubble plot showing the fold change of differentially abundant common **(A)** and rare **(B)** taxa associated with health disparities (control and USD phenotypes) in urine samples. Selection of taxa is defined using DESeq2 differential abundance analysis with a false discovery rate-corrected *p*-value <0.05. Taxa are listed as the number of ASVs within the lowest assigned taxonomy as a means to elucidate the most important taxa associated with USD.

In gut microbiota, rare taxa such as Christensenellaceae, Clostridia, Lachnospiraceae, and Oscillospirales exhibited the greatest number of ASVs significantly enriched in the healthy control group, while common taxa such as Bacteroidales and Lachnospiraceae, along with rare taxa such as Clostridia and Bacteroides, exhibited the greatest number of ASVs significantly enriched in the USD group (Figure 6; Supplementary Material S2, S3). In urine samples, rare taxa such as *Actinomyces*, *Anaerococcus*, *Bacteroides*, *Corynebacterium*, and *Sphingomonas* and common taxa such as *Corynebacterium* exhibited the most number of ASVs significantly enriched in the healthy control group, while common taxa such as *Anaerococcus* and *Corynebacterium* exhibited the most number of ASVs significantly enriched in the USD group (Figure 7; Supplementary Material S4, S5).

## Discussion

### Rare phylotypes represent major contributions to microbiome structure and their association with diseases

One of the common first steps in microbiome analyses is to remove technical artifacts, contaminants, and rare taxa (Cao et al., 2021). The removal of rare taxa eliminates a large number of species and simplifies data to make it easier to comprehend. However, the data presented here clearly show that rare phylotypes make considerable contributions to the structure of microbial communities (Figure 4) and are significantly associated with disease phenotypes (Figure 5). Across all sample types, 90% or more of the individual ASVs were rare. As such, the arbitrary removal of rare taxa likely masks biologically important phylotypes within microbial communities, which likely has important implications for understanding the microbiome function (D Ainsworth et al., 2015). In fact, it is recognized that rare microbial species play a major role in species turnover and conservation of phylogenetic elements, which often makes these keystone species vital for the functioning of multiple environments (Jousset et al., 2017).

The results of alpha diversity (Figure 1C) and taxonomic profiling (Supplementary Figures S3–S8) indicate that stool is the most phylogenetically diverse sample type, while the urinary tract harbors unique niches between the upper and lower poles (Figure 2). Microbiome composition differences in the sample type are driven by both common and rare phylotypes (Figure 2). Of particular importance, while all sample types were dominated by rare taxa, we saw a clear shift of increasing rarity from stool to urine and then to stone. Previous studies have found that the shift toward rare taxa in ecosystems are indicative of resource scarcity (Wassen et al., 2005; Farrer and Suding, 2016; Bickel and Or, 2021) and that resource scarcity drives spatial organization and development of microniches (Mitri et al., 2016). Previous studies of the stone microbiome have revealed clear spatial microniches and microbial activities within the stone matrix (Saw et al., 2021). As such, the results reported here likely reflect the development of microniches, particularly in kidney stones and the lower urinary tract, where resources are far scarcer than those for the gastrointestinal tract, which shows a steady influx of resources through host diet.

The study’s findings have important implications for understanding the role of the microbiome in health and diseases, especially in kidney stone disease. Our results show that both the common and rare microbiomes of urine as well as stool samples are associated with USD (Figure 5), which is consistent with previous whole-microbiome studies (Kachroo et al., 2021). The identification of specific phylotypes associated with kidney stone disease in stool and urine samples (Figures 6, 7) provides a basis for further investigations into the pathogenesis of the disease. It is unclear from the current data whether common, rare, or any bacteria play a direct role in lithogenesis. However, if bacteria directly contribute to lithogenesis by promoting local mineralization in microniches of the kidneys, potential therapeutic strategies may need to focus on the primary resources that support these bacteria. If more abundant bacteria are the primary drivers of lithogenesis, this task would be simple and could target one or a few substrates for growth. If, however, our data suggest that rare taxa are not only the dominant bacteria found collectively but also vital for lithogenesis, it may be more difficult to prevent since microniches specifically arise with resource limitation. This is particularly relevant for antibiotic use as antibiotics are unlikely to eradicate all bacteria in all microniches where stones form and may in fact promote the growth of surviving, antimicrobially resistant, and lithogenic bacteria. In fact, numerous studies have independently found significant associations between previous antibiotic use and kidney stone formation (Tasian et al., 2018; Ferraro et al., 2019; Zampini et al., 2019). Future studies will need to tease apart the hypotheses of common vs*.* rare taxa and the role of antibiotic use in promoting lithogenesis.

**FIGURE 8:**
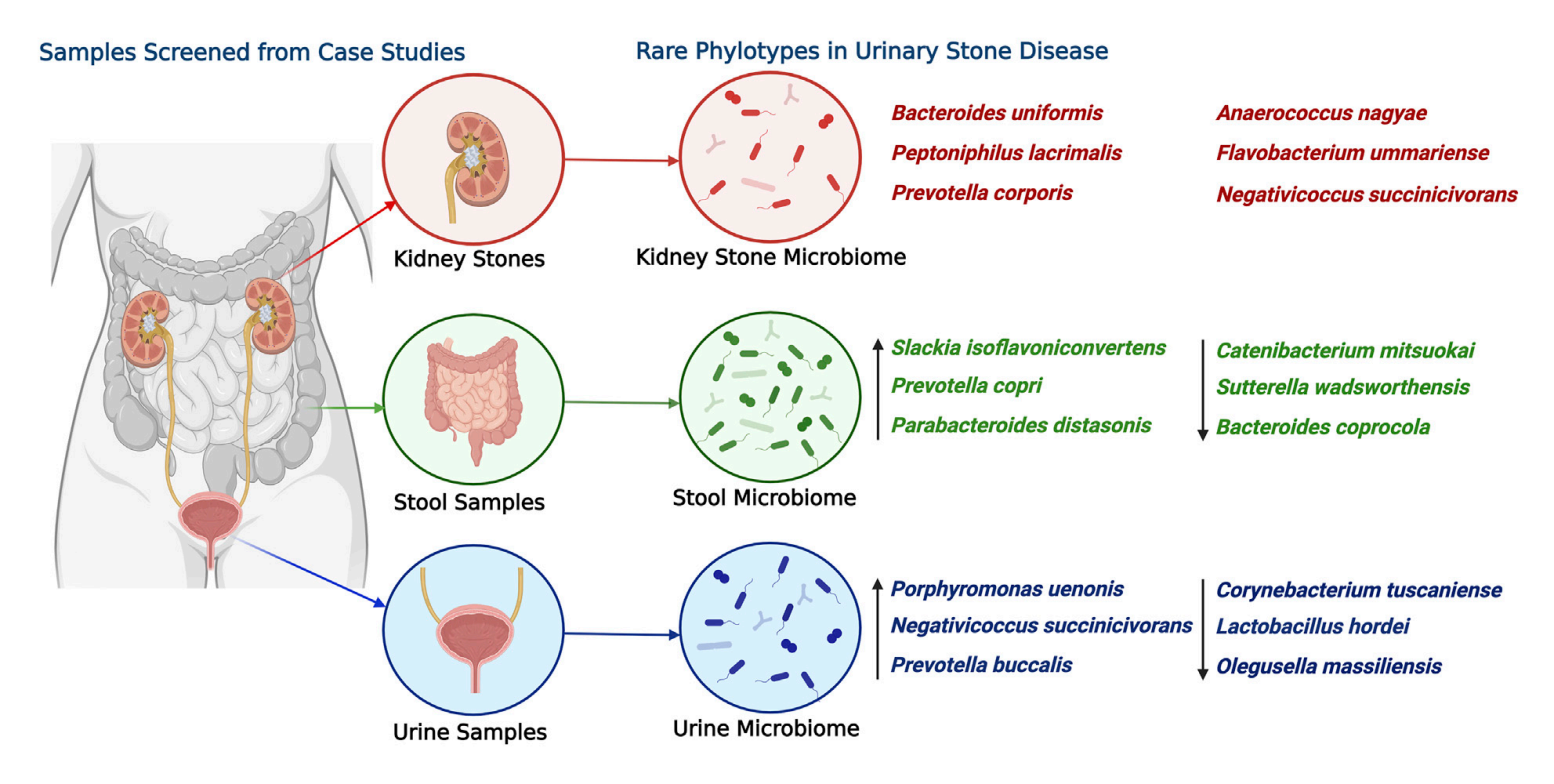
Most abundant rare phylotype species found in the kidney stone, urine, and stool samples associated with USD. The ASVs are ranked by the order of descending abundance. The relative abundance and distribution of these species are depicted to shed light on potential health disparity patterns. Emphasis is placed on the six most abundant ASVs from kidney stones, with arrows used to point out the three most significantly abundant ASVs from urine and stool samples. This illustration was created using “BioRender,” and a publishing license for this graphic has been obtained.

Overall, the study highlights the need for a comprehensive understanding of the microbiome structure and composition, including rare phylotypes (Figure 8), to gain insights into the role of the microbiome in health and diseases. The findings of the study provide a basis for further investigations into the microbial ecology of different sample types and their potential roles in disease pathogenesis. This study also underscores the significance of rare taxa in microbiome analysis and their potential impact on microbial composition and disease outcomes. While excluding rare taxa aids in maintaining data quality by minimizing sequencing artifacts and errors, it is imperative to reassess their inclusion following rigorous quality control measures that are commonly implemented in contemporary bioinformatic pipelines. With such measures, investigations can deepen our understanding of the role of rare taxa and expand our knowledge of the intricate dynamics of the microbiome.

## Summary

It is clear that kidney stones, gut, and midstream urine are all dominated by rare phylotypes with a greater bias toward rarity in urine and stones. It appears that rare taxa are especially important in the microbiome structure in stones and to a lesser extent in urine, whereas stool appears to cluster around common phylotypes. Both common and rare phylotypes contribute to the differences observed by the sample type and USD status. The shift toward rarity may be indicative of resource scarcity in the environment and is likely to be biologically meaningful. Our data suggest that the consideration of rare taxa may be vital in developing therapeutic strategies to disrupt the kidney stone matrix or other microbially derived phenotypes. As such, it is vital for microbiome studies to maintain biologically important rare taxa in analyses in order to understand the potential function and/or drivers of disease.

## Data Availability

Data used for the study are available at the sequence read archive under accession numbers SRP140641, SRP140933, SRP066940, SRP103884, SRP125171, and SRP125191. A reference library with 16S rRNA gene sequences for stone associated microbiome has been created and scripts used for analysis can be found at https://github.com/amill017/rare_phylotype.

## Supplementary material

SUPPLEMENTARY MATERIAL S1 Sample description and associated metadata for all the samples under study.

SUPPLEMENTARY MATERIAL S2 List of differentially abundant common stool phylotypes in health disparity.

SUPPLEMENTARY MATERIAL S3 List of differentially abundant rare stool phylotypes in health disparity.

SUPPLEMENTARY MATERIAL S4 List of differentially abundant common urine phylotypes in health disparity.

SUPPLEMENTARY MATERIAL S5 List of differentially abundant rare urine phylotypes in health disparity.

SUPPLEMENTARY FIGURE S1 Histogram showing the distribution of ASV count abundances in each sample type. **(A)** Histogram of ASV counts detected in stone samples; **(B)** histogram of ASV counts detected in stool samples; **(C)** histogram of ASV counts detected in urine samples.

SUPPLEMENTARY FIGURE S2 Histogram showing the distribution of taxon prevalence in each sample type. **(A)** Histogram of taxon counts detected in stone samples; **(B)** histogram of taxon counts detected in stool samples ; **(C)** histogram of taxon counts detected in urine samples.

SUPPLEMENTARY FIGURE S3 Bar plot showing the relative abundance of common and rare phylotypes at the phylum level in stone samples. **(A)** Common phylotypes in phylum-level taxa observed in stone samples. **(B)** Rare phylotypes in the phylum-level taxa observed in stone samples.

SUPPLEMENTARY FIGURE S4 Bar plot showing the relative abundance of common and rare phylotypes at the genus level in stone samples. **(A)** Common phylotypes in genus-level taxa observed in stone samples. **(B)** Rare phylotypes in genus-level taxa observed in stone samples.

SUPPLEMENTARY FIGURE S5 Bar plot showing the relative abundance of common and rare phylotypes at the phylum level in stool samples. **(A)** Common phylotypes in phylum-level taxa observed in stool samples. **(B)** Rare phylotypes in phylum-level taxa observed in stool samples.

SUPPLEMENTARY FIGURE S6 Bar plot showing the relative abundance of common and rare phylotypes at the genus level in stool samples. **(A)** Common phylotypes in genus-level taxa observed in stool samples. **(B)** Rare phylotypes in genus-level taxa observed in stool samples.

SUPPLEMENTARY FIGURE S7 Bar plot showing the relative abundance of common and rare phylotypes at the phylum level in urine samples. **(A)** Common phylotypes in phylum-level taxa observed in urine samples. **(B)** Rare phylotypes in phylum-level taxa observed in urine samples.

SUPPLEMENTARY FIGURE S8 Bar plot showing the relative abundance of common and rare phylotypes at the genus level in urine samples. **(A)** Common phylotypes in genus-level taxa observed in urine samples. **(B)** Rare phylotypes in genus-level taxa observed in urine samples.
