# Supplementary figures and images for: Rare phylotypes in stone, stool, and urine microbiomes are associated with urinary stone disease
Source: Front Mol Biosci. 2023 Aug 4;10:1210225. doi: 10.3389/fmolb.2023.1210225 (PMC10436313; doi:10.3389/fmolb.2023.1210225)

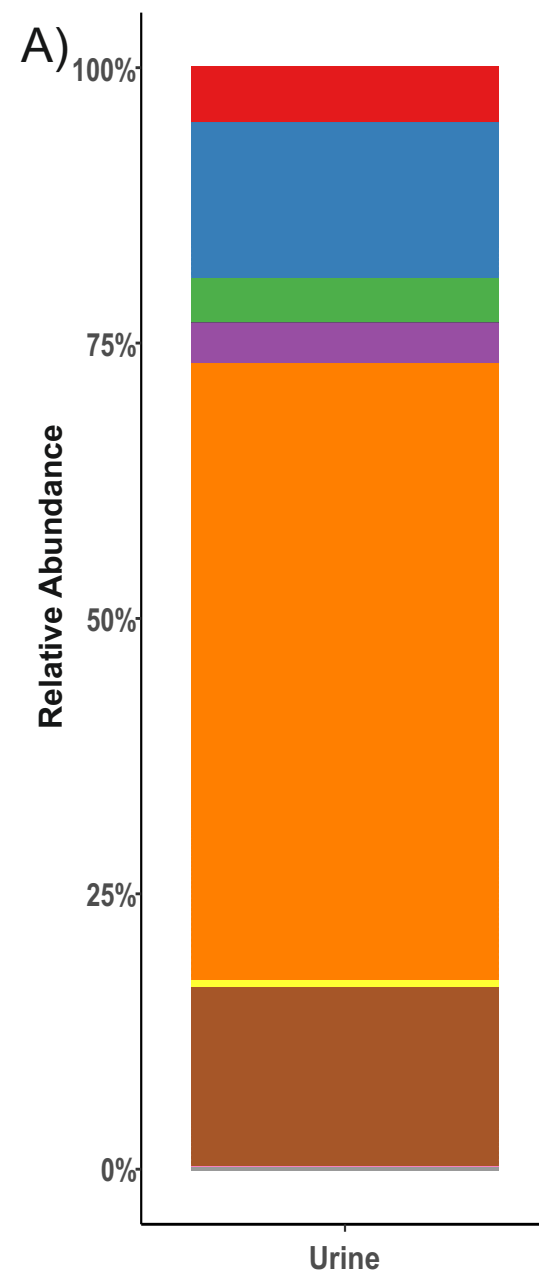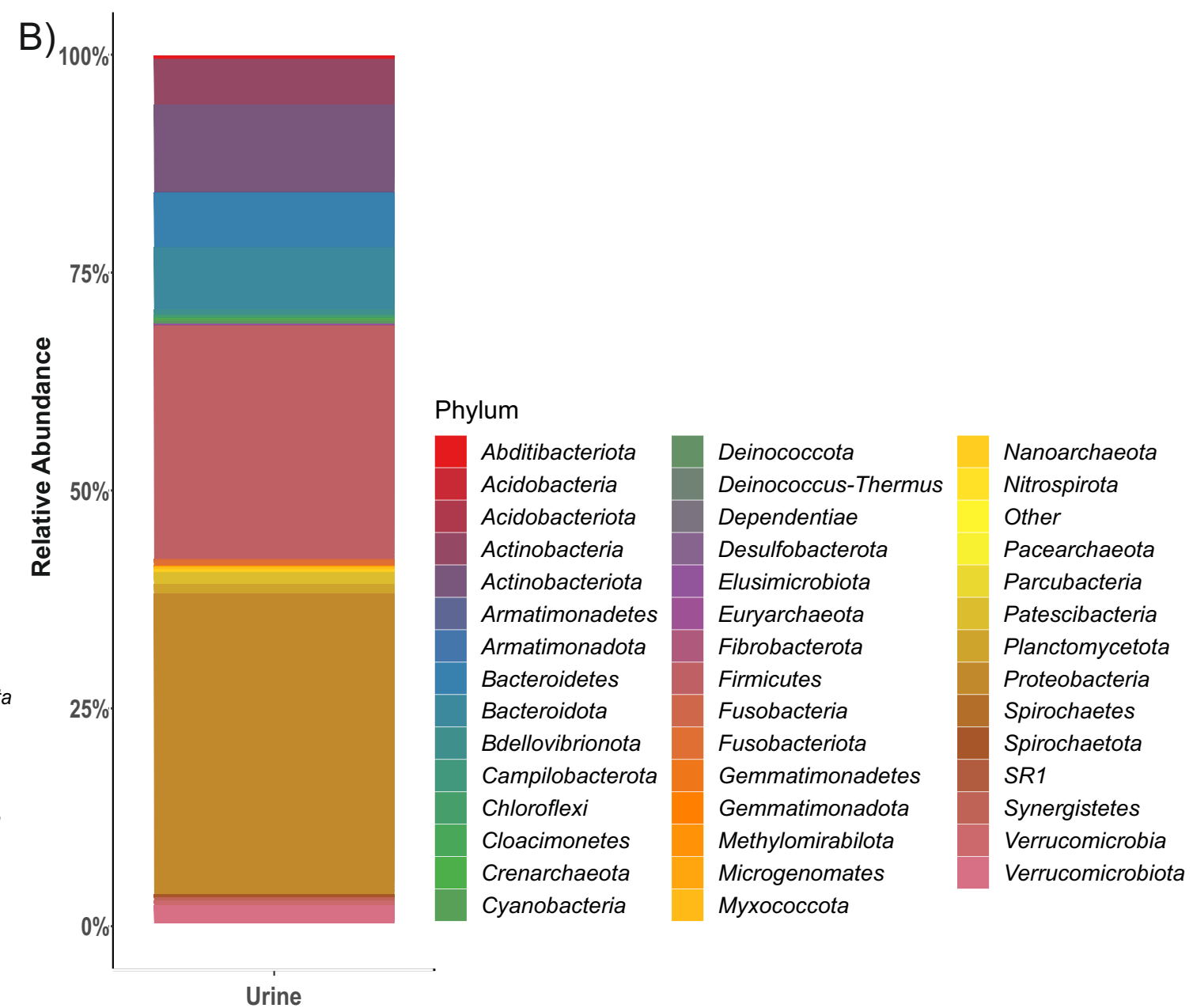

Supplement: Supplementary file 1 [file DataSheet7.PDF]

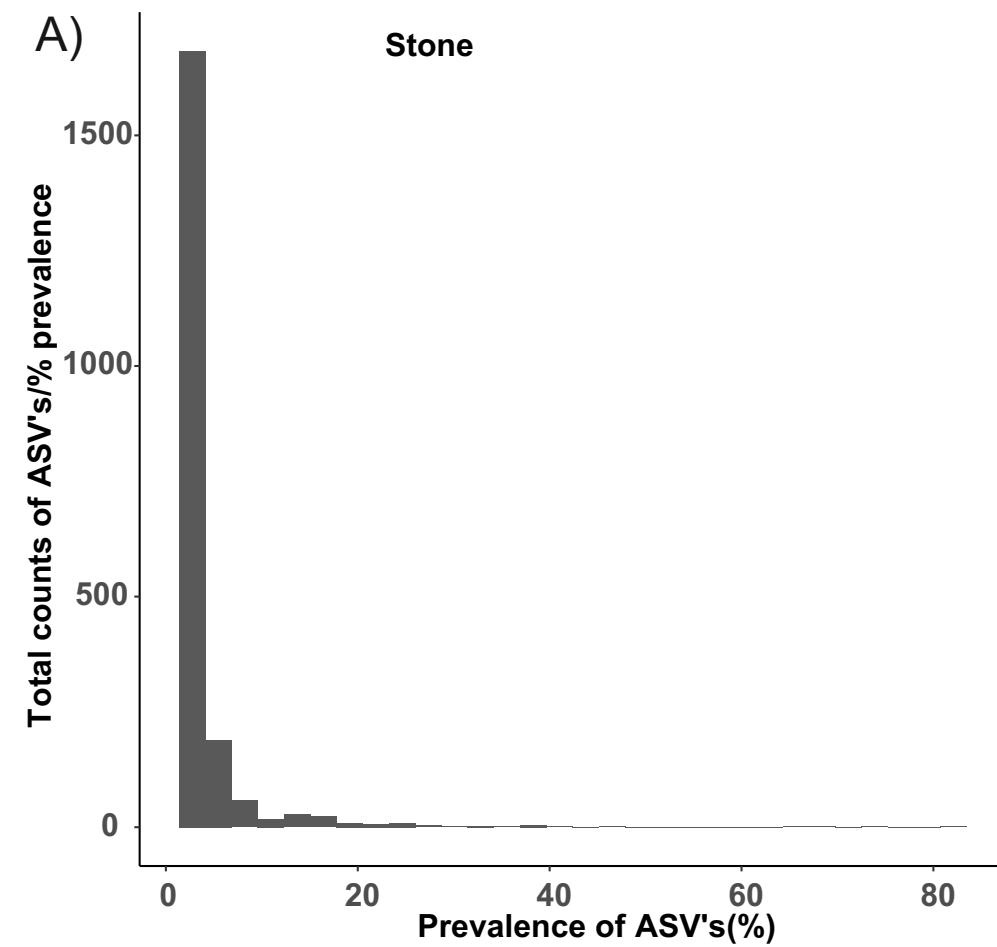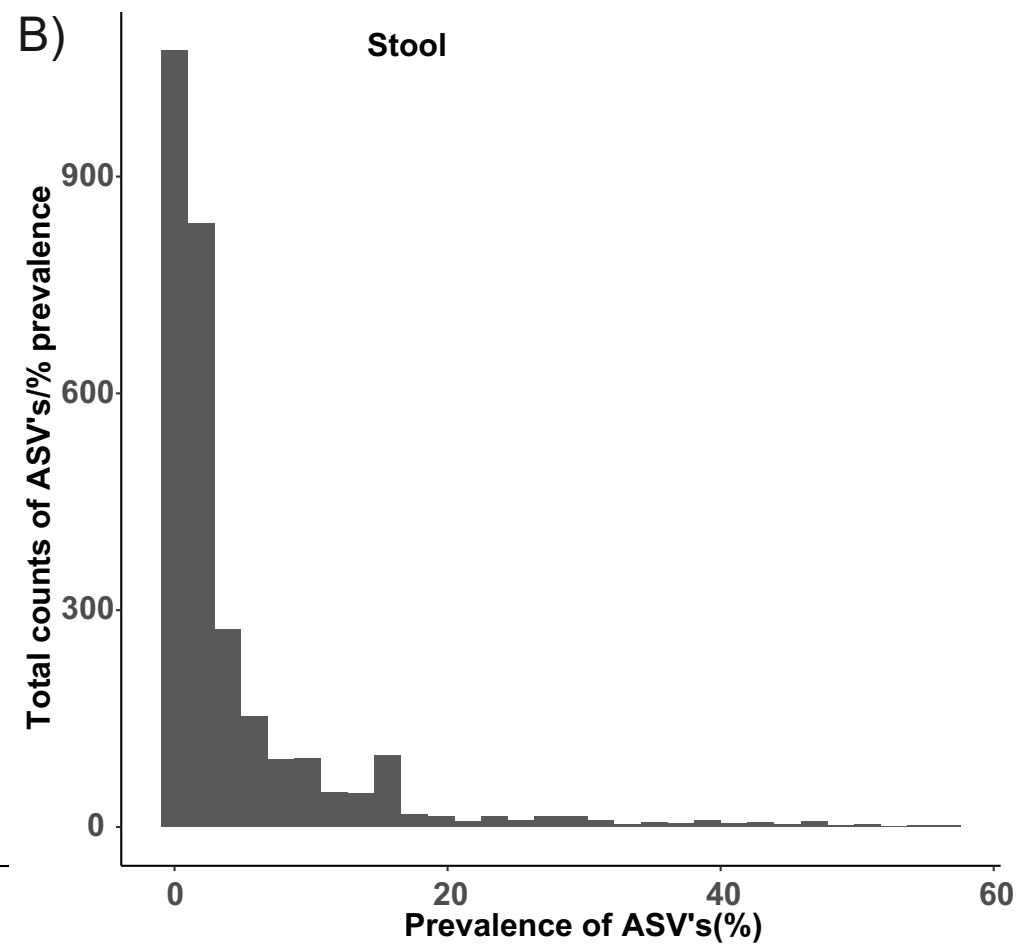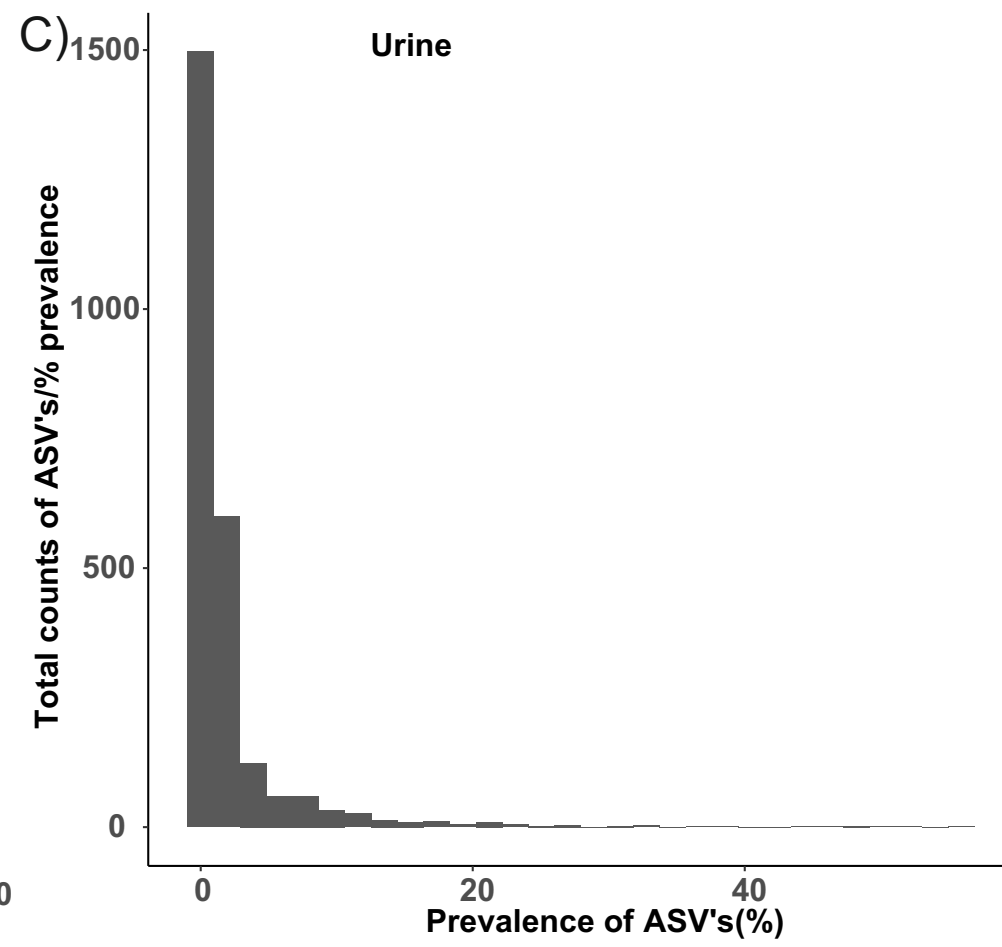

Supplement: Supplementary file 2 [file DataSheet2.PDF]

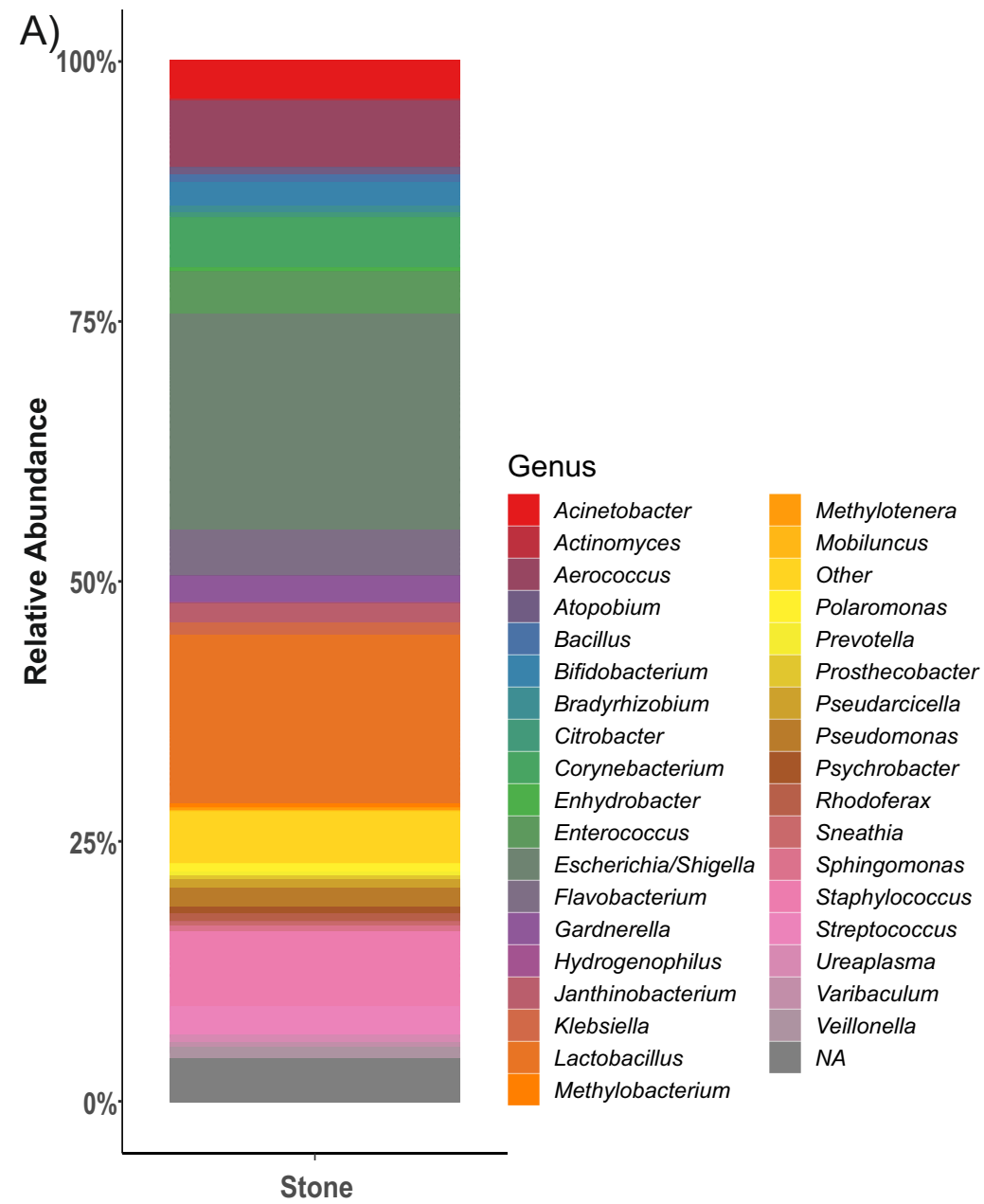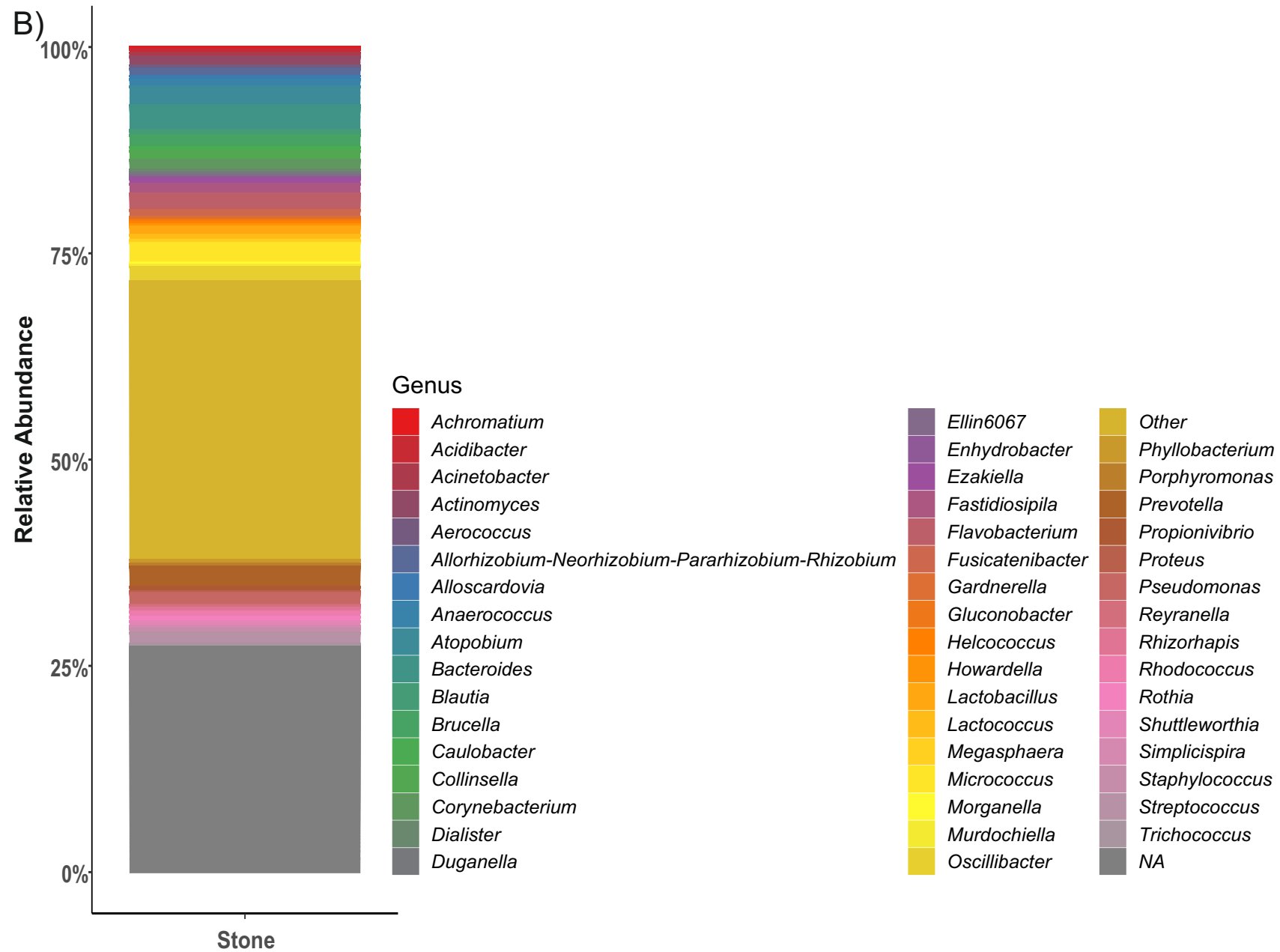

Supplement: Supplementary file 4 [file DataSheet4.PDF]

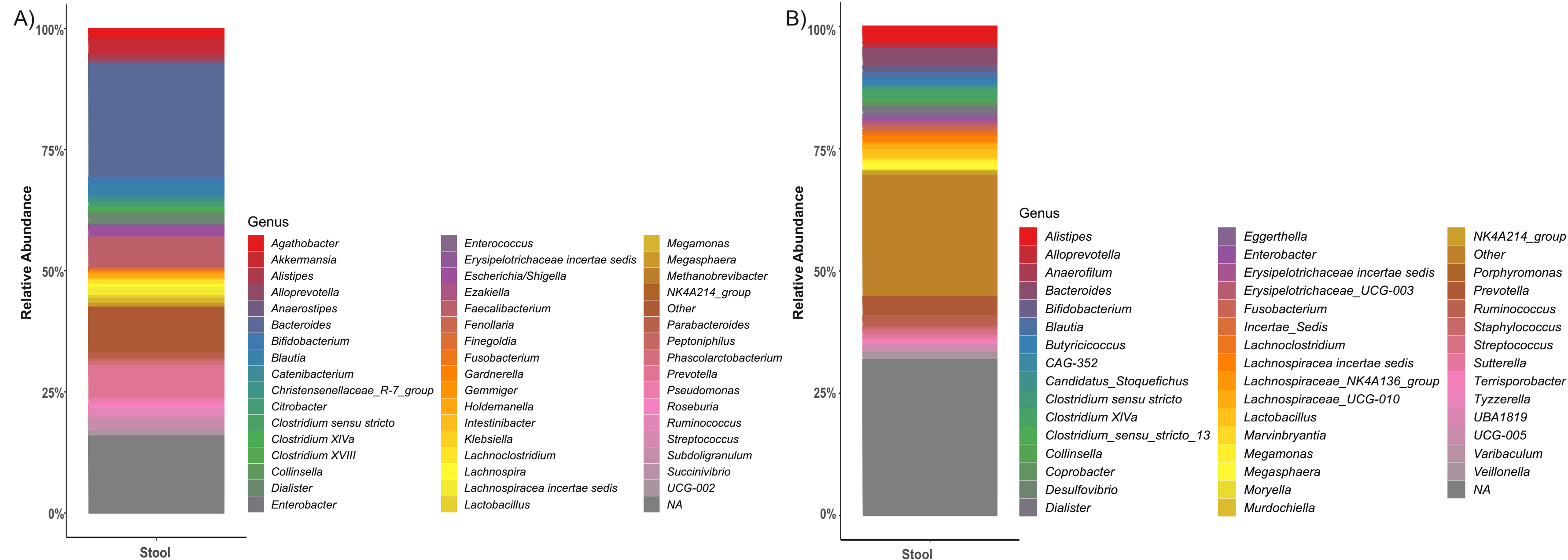

Supplement: Supplementary file 5 [file DataSheet6.PDF]

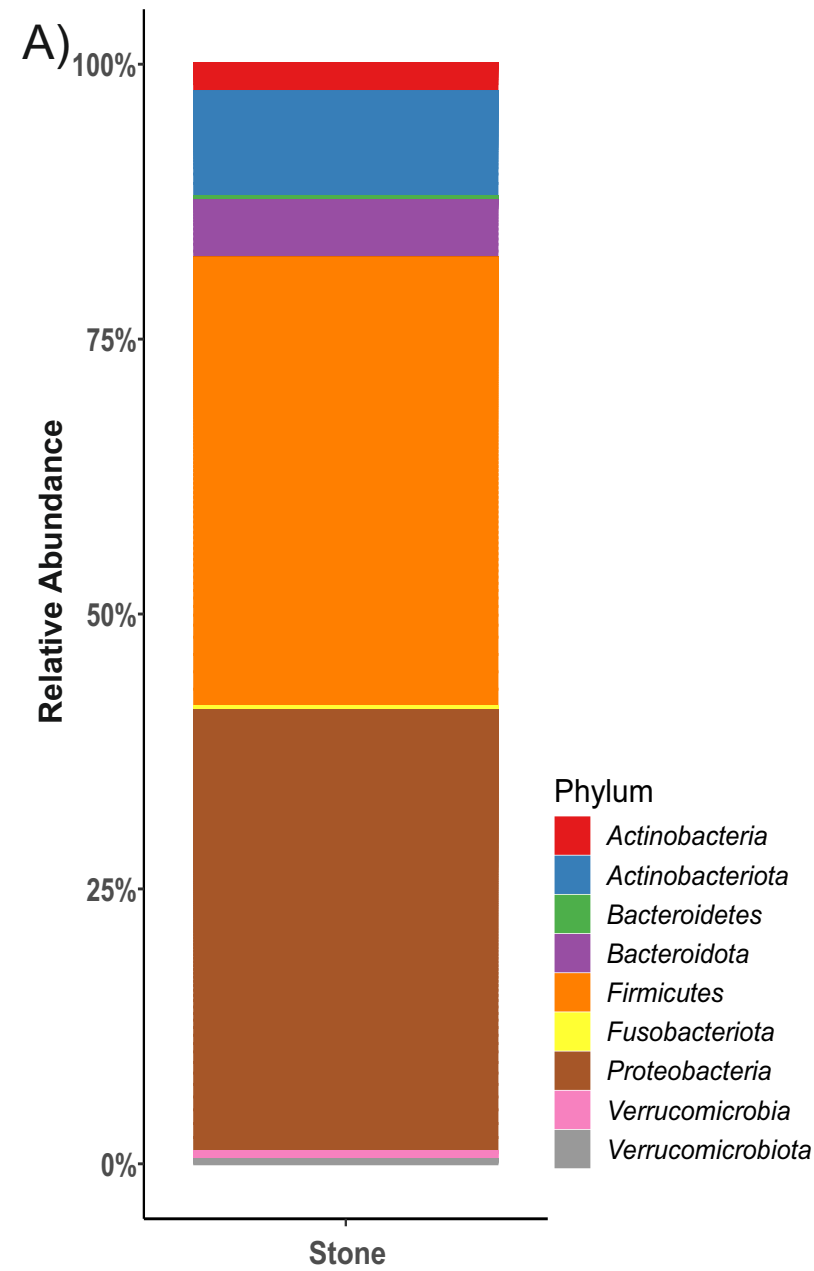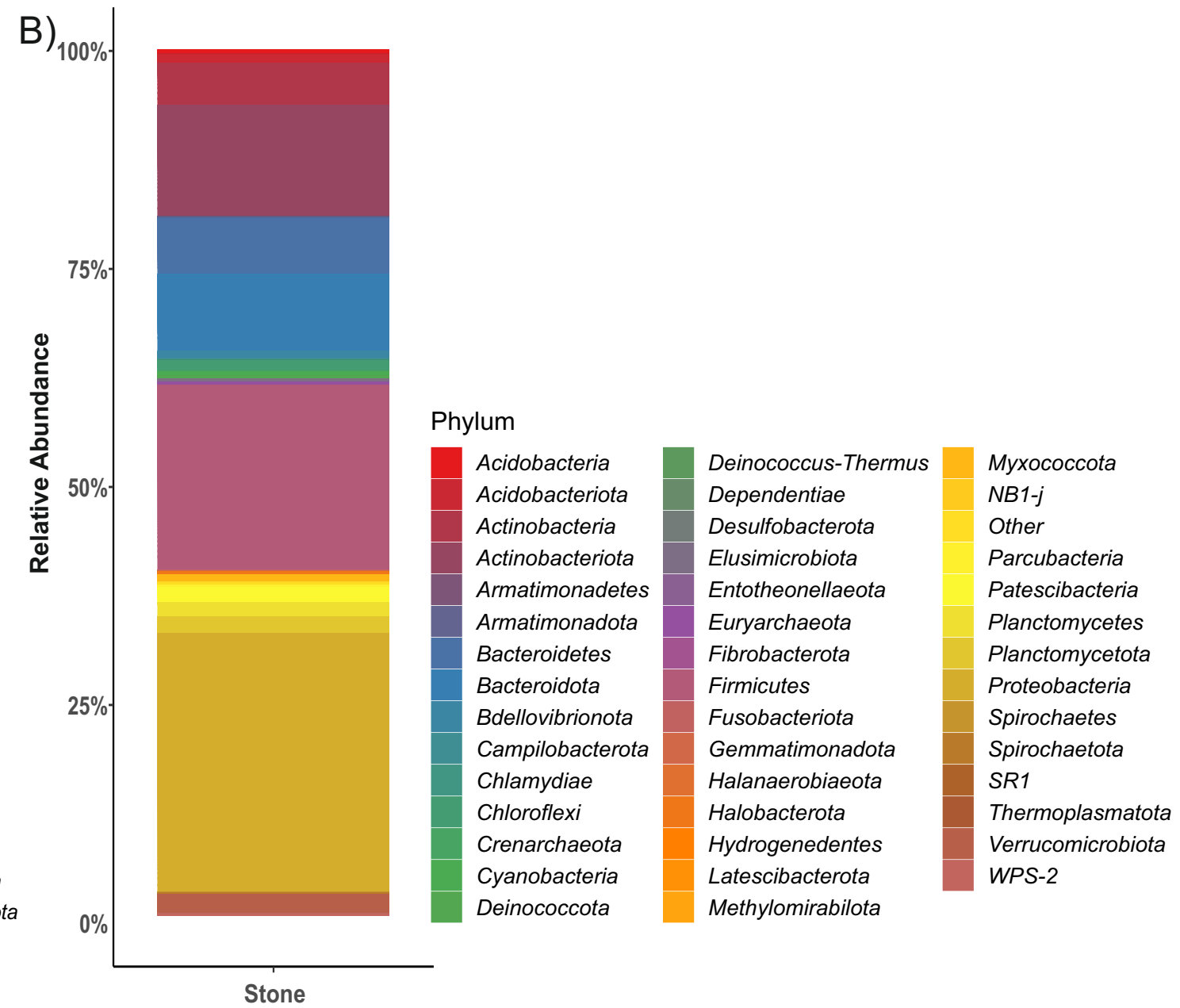

Supplement: Supplementary file 7 [file DataSheet3.PDF]

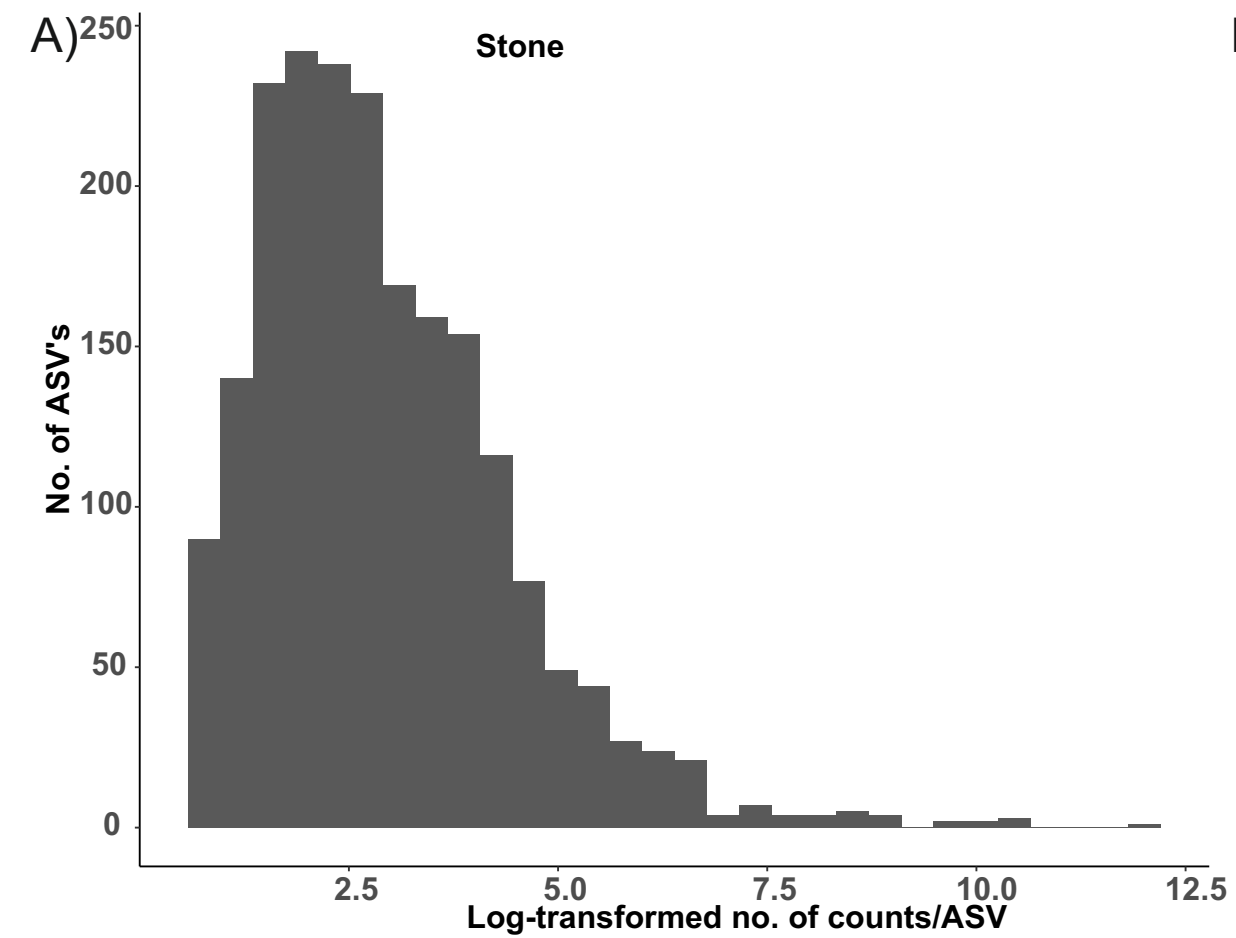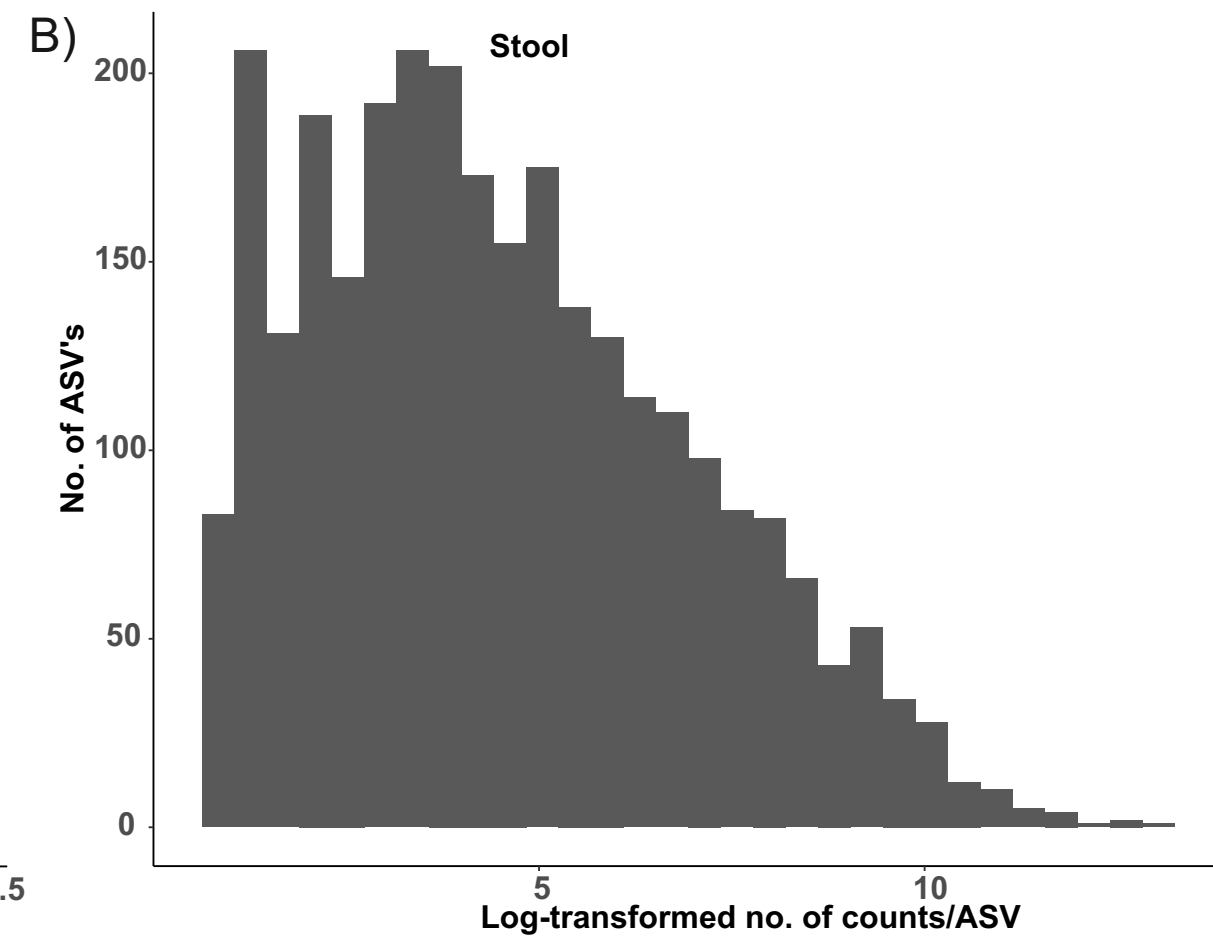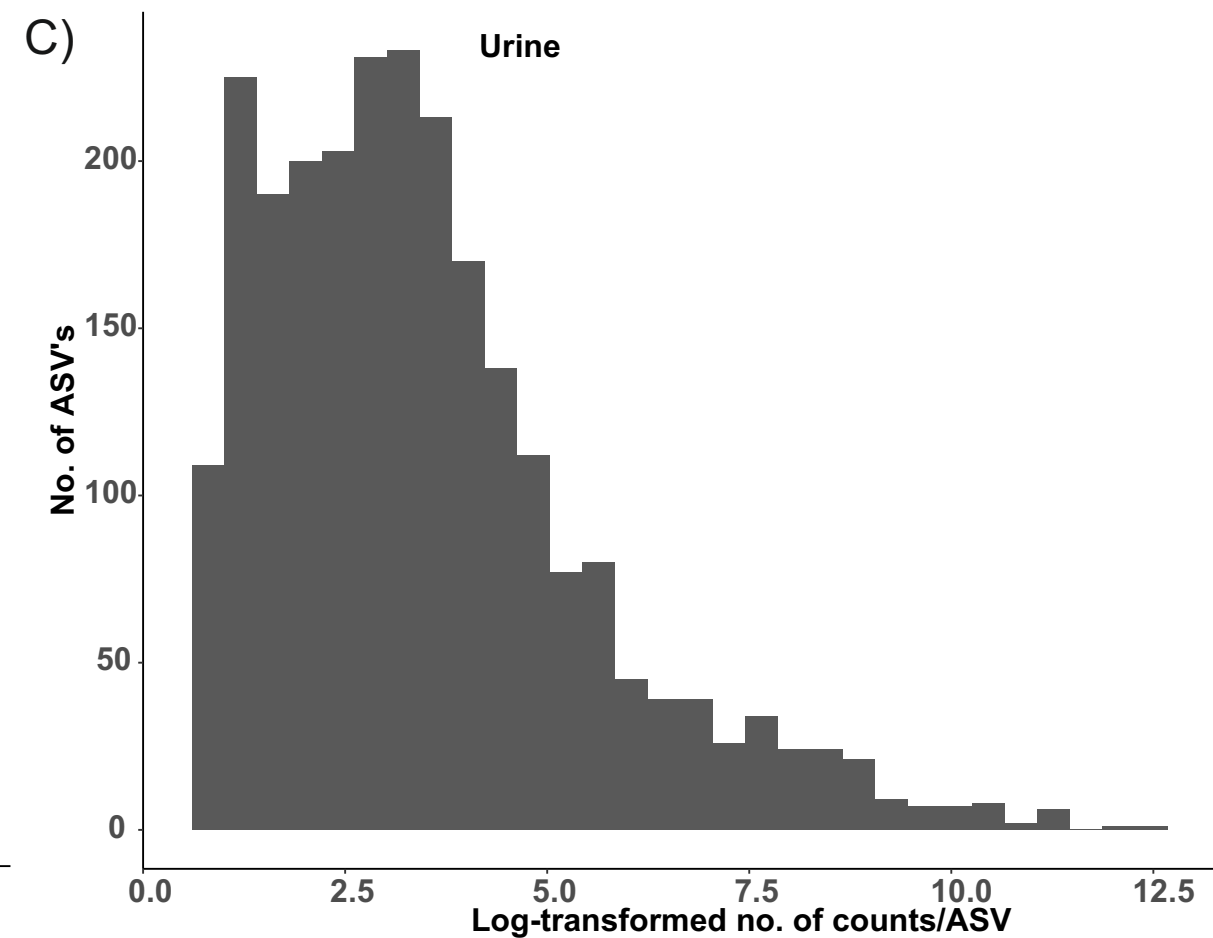

Supplement: Supplementary file 8 [file DataSheet1.PDF]

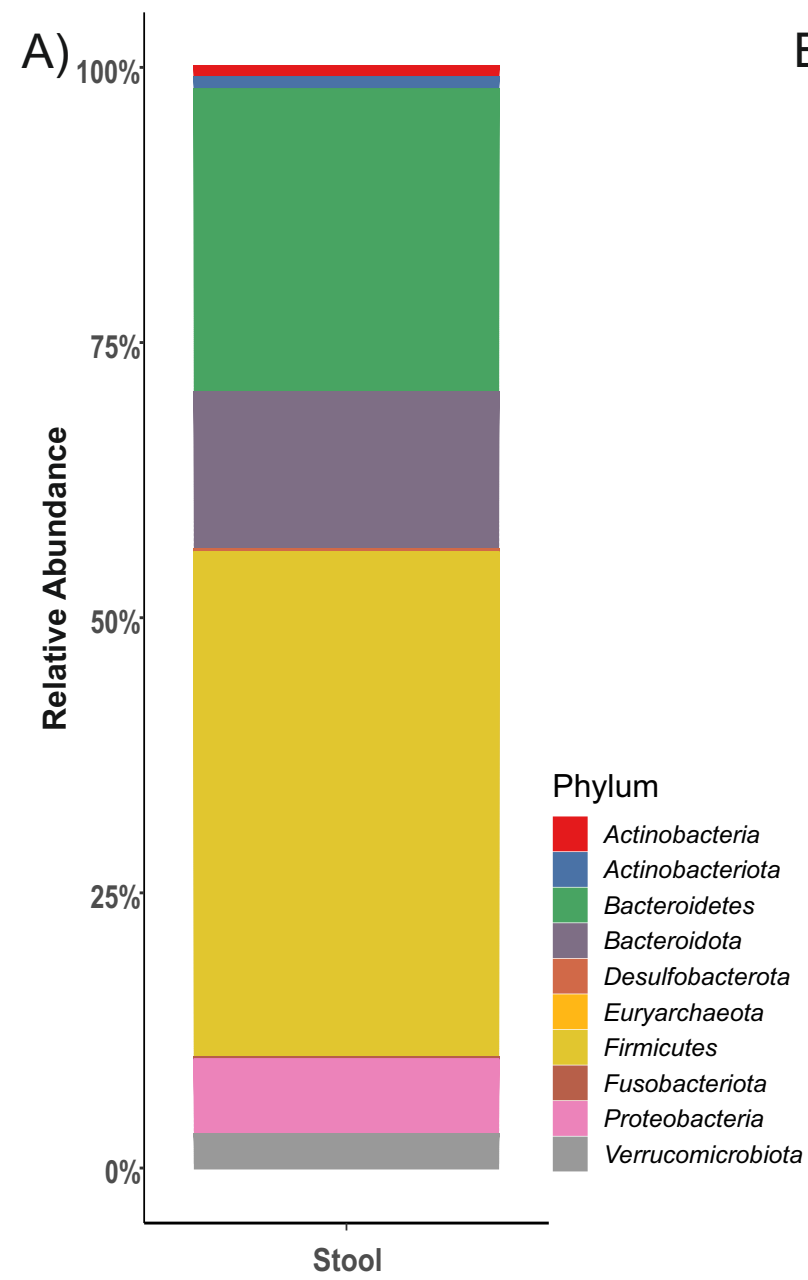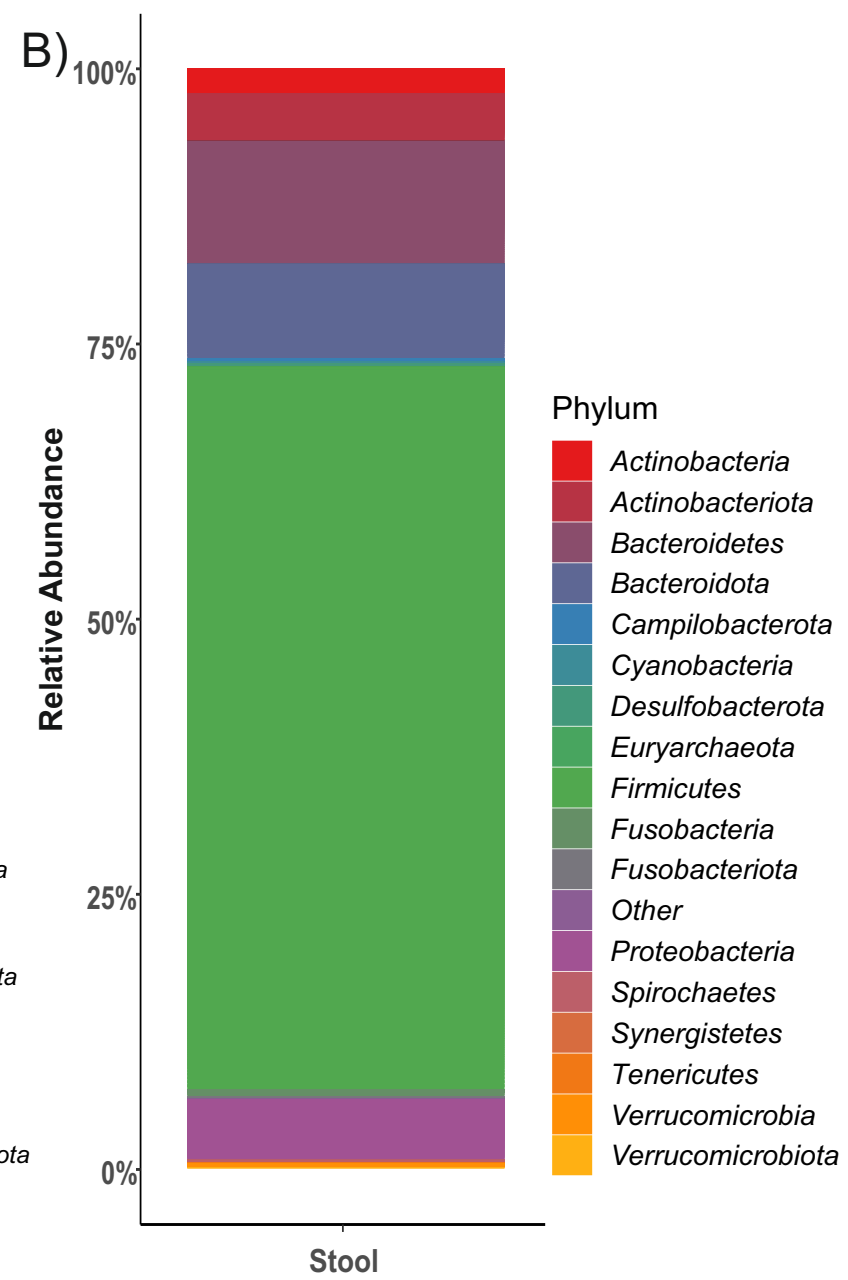

Supplement: Supplementary file 9 [file DataSheet5.PDF]

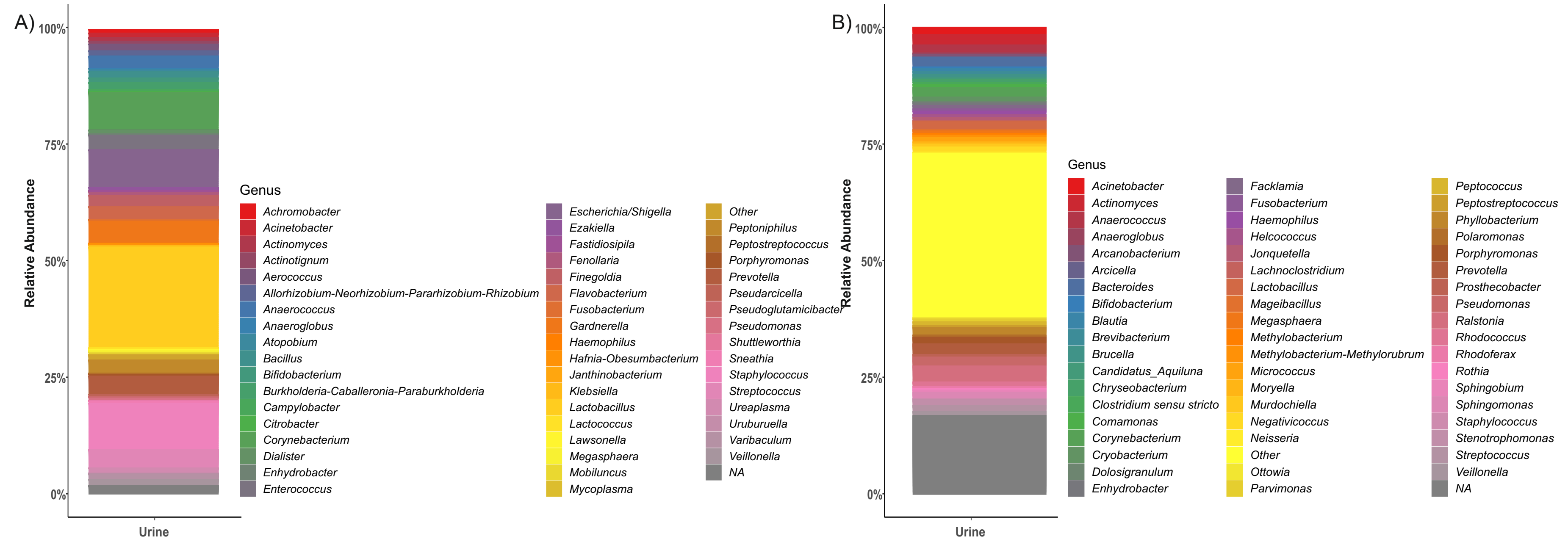

Supplement: Supplementary file 12 [file DataSheet8.PDF]
